# Prevalence of Patients Affected by Fibromyalgia in a Cohort of Women Underwent Mammography Screening

**DOI:** 10.3390/healthcare9101340

**Published:** 2021-10-08

**Authors:** Gianluca Gatta, Daniele La Forgia, Annarita Fanizzi, Raffaella Massafra, Francesco Somma, Maria Paola Belfiore, Daniela Pacella, Salvatore Cappabianca, Antonio Alessandro Heliot Salvia

**Affiliations:** 1 Dipartimento di Medicina di Precisione Università Della Campania “Luigi Vanvitelli”, 80127 Napoli, Italy; gianluca.gatta@unicampania.it (G.G.); mariapaola.belfiore@unicampania.it (M.P.B.); salvatore.cappabianca@unicampania.it (S.C.); antoniosalvia89@gmail.com (A.A.H.S.); 2 IRCCS Istituto Tumori “Giovanni Paolo II”, 70124 Bari, Italy; a.fanizzi@oncologico.bari.it (A.F.); r.massafra@oncologico.bari.it (R.M.); 3 ASL NA1 Centro–Ospedale del Mare, 80127 Napoli, Italy; fra1585@hotmail.it; 4 Dipartimento Sanità Pubblica, Università degli Studi di Napoli “Federico II”, 80127 Napoli, Italy; daniela.pacella@unina.it

**Keywords:** fibromyalgia, women, diagnostic imaging

## Abstract

Fibromyalgia is a widespread condition which is currently underdiagnosed; therefore we conceived this study in order to assess whether a diagnostic suspicion may be assumed during widespread screening procedures, so that patients for which a reasonable diagnostic suspicion exist may be redirected towards rheumatologic evaluation. We analyzed a sample of 1060 patients, all of whom were female and undergoing standard breast cancer screening procedures, and proceeded to evaluate the level of pain they endured during mammographic exam. We also acquired a range of other information which we related to the level of pain endured; we suggested a rheumatologic examination for those patients who endured the highest level of pain and then we evaluated how many patients in this subgroup were actually diagnosed with fibromyalgia. Out of the 1060 patients who participated to our study, 139 presented level 4 pain intensity; One patient did not go for rheumatologic examination; the remaining 138 underwent rheumatologic evaluation, and 50 (36%, 28–44, 95% CI) were diagnosed with fibromyalgia. Our study shows that assessing the level of pain endured by patients during standard widespread screening procedures may be an effective asset in deciding whether or not to suggest specialist rheumatologic evaluation for fibromyalgia.

## 1. Introduction

Fibromyalgia (FM) is a complex systemic pathology characterized by generalized musculoskeletal pain, sleep disturbance, stiffness, fatigue, and psychological problems [1,2]. FM is defined by the American College of Rheumatology (ACR) [3] as a chronic musculoskeletal pain syndrome whose etiology is currently unknown, characterized by widespread pain for more than 3 months and soreness in at least 11 out of 18 tender points.

Diagnostic criteria were modified by the ACR in 2010 and 2011, firstly shifting the diagnostic focus away from tender points and towards a clearer and more specific variety of symptoms, then allowing for the possibility of self-reported diagnosis in a research setting, as well as adding a fibromyalgia severity score [3].

Cognitive difficulties, while not commonly assessed at diagnosis, are also common in FM. These include so called “Fibro Fog,” or dyscognition, defined as cognitive dysfunction characterized by memory lapses, confusion, as well as impairing concentration, planning, and organization [4]. Fibro Fog is experienced by 76.4–82.5% of patients with FM [5], yet cognitive impairment was only added to the ACR diagnostic criteria in 2010 [3]. Major depression has been found to be 20–60% more prevalent among patients with FM when compared to the general population [6].

The etiology of FM is unknown, and it is likely multifactorial. First-degree relatives of patients with FM are 8.5 times more likely to have this disorder than the general population [7]. However, genetic factors associated with FM are unknown. It has been suggested that serotonin- and dopamine-related genes may play a role in the pathogenesis of FM [8].

The strongest evidence points to dysregulation of pain modulation, based upon the results of experimentally induced pain testing yielding heightened processing and attenuated inhibitory process of noxious stimuli [9,10], as well as imaging studies showing increased neural response to pain.

Patients with FM have a three-times-higher concentration of substance P in the cerebrospinal fluid [11]. Activation of the N-methyl-d-aspartate receptor (NMDAR) is also increased in FM patients. Substance P modulates the responsiveness of the NMDAR to glutamate, which consequently leads to temporary central sensitization and temporal summation in otherwise healthy individuals [12,13]. It has been demonstrated that FM patients’ serotonin levels in the serum are reduced and inversely correlated with pain threshold [14,15]. Combined dysfunctional neurotransmitter systems, such as low serotonin and high substance P levels, can produce more pain than either abnormality on their own and be responsible for the onset of FM [16,17].

Research has demonstrated that FM patients have a lower level of dopamine, which plays a central role in painful conditions modulating pain perception and natural analgesia within supraspinal regions and the spine [18,19]. It was found that several serum pro-inflammatory cytokines, such as tumor necrosis factor (TNF)-α and interleukin, are involved in the generation of symptoms in FM, including sleep disturbances, fatigue, and myalgia [8,20,21,22].

Some studies have shown a relationship between smoking habit and FM [23].

Prevalence rates of FM have been found to vary between 0.2–4.7% [24]. Among women, prevalence rates range between 2.4–6.8% [24], with about a 9:1 female-to-male prevalence ratio [25], although one study found slightly less of a distinct gender difference [26]. Interestingly, in one recent study conducted by Wolfe and colleagues [27] among a sample of 2445 adults, no significant gender difference in FM prevalence rates was found. One explanation concerning this disparity could be the changing diagnostic criteria. The reliance of the original ACR criteria on tender points may have resulted in higher FM rates among women, as women were found to have more tender points than men [3]. Since Wolfe and colleagues [27] utilized the modified ACR criteria, which rely on tender points to a lesser degree, the gender ratios may have become more proportionate.

There is currently no mass screening program for the detection of fibromyalgia; we decided to evaluate the possibility of assuming the diagnostic suspicion of pain using well-established mass screening procedures (breast cancer mammography screening programs, in our case).

**Figure 1:**
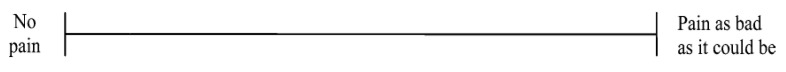
Visual Analogue Scale (VAS).

**Figure 2:**
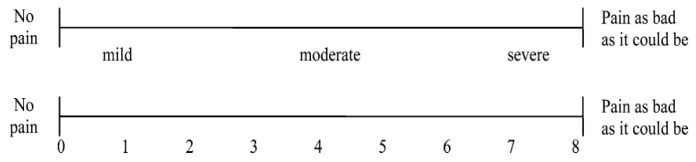
Examples of Graphic Rating Scale (GRS).

In the literature, there are a series of reference scales validated for diagnosing pain [28], including the VAS (visual analogue scale) andNRS (numeric pain rating scale) one-dimensional scales, which are certainly simple and rapid but poor in separate information regarding the extent of the pain. These scales identify two extremes consisting of the absence of pain and the maximum possible pain by identifying intermediate situations expressed by numerical values(Figure 1 and Figure 2).

Numerical rating scales have shown high correlations with other pain assessment tools in several studies [29,30]. The feasibility of its use and good conformity [31,32] has also been demonstrated. Since it is easily possible to administer NRS verbally, it can be used in telephone interviews [33].

More complex scales (e.g., McGill Pain Questionnaire (MPQ)) help to better frame not only the presence/absence of pain but also the impact on the amount of life and daily activities; there are also dedicated scales to detect the possible presence of neuropathic pain (e.g., DN 4 douleur neuropatique 4), or the risk of addiction in patients undergoing opioid therapy (ORT, opioid risk tool, recently also available in Italian) [28].

It has been shown that pain intensity can be reported quite easily by most patients and that several methods of pain intensity measurement have shown a high intercorrelation [29,34]. However, many factors such as the social situation, the work situation and environment and the history of a previous injury can influence the perception of pain and show great differences between individuals [28].

The aim of this study is, therefore, to investigate whether a link can exist between the level of pain experienced by female patients while undergoing standard mammographic cancer screening procedures and an eventual diagnosis of FM; a subordinate aim was to assess the association between the level of pain endured by the patients and a range of other factors gathered with a questionnaire filled by the patients.

## 2. Materials and Methods

This prospective study was performed between June 2017 and July 2020.

We analyzed a sample of 1060 patients, all females and all Caucasian, to be subjected to standard breast cancer screening procedures, including mammography and 3D prone ecography with the Sofia system (Hitachi, Tokyo, Japan); we excluded from our study those patients who had previously received breast surgical interventions, those whose breasts were affected by benign pathologies, those who were breastfeeding and those who were affected by other rheumatologic pathologies.

Our study proceeded in two distinct phases: during the first phase (round 1) our patients, after having undergone their mammographic exam, were interviewed in order to acquire the following information: age, level of pain on a semiquantitative basis (1–4, where 1 corresponds to “little to no pain”, 2 to “moderate pain”, 3 to “significant pain” and 4 to “severe or unbearable pain”), smoking habit and number of cigarettes/day, coffee drinking habit and number of coffee cups/day, number of children, previous breastfeeding, level of perceived psychological stress, educational qualification, annual income, breast density along with ACR BIRADS classification. We did not consider breast size as a factor as there is no significant evidence of a relation between size and breast pain; in fact, looking at the medical literature, there isnot enough research on the subject in order to demonstrate the contrary [35].

**Figure 3:**
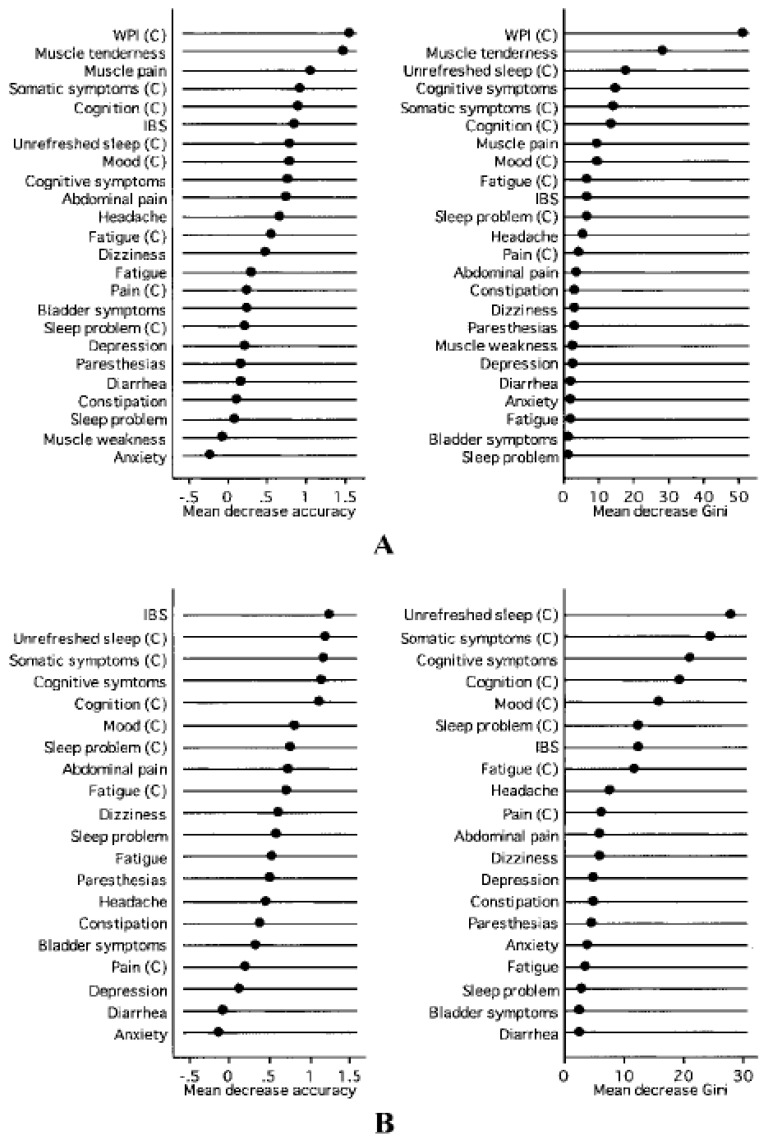
Symptoms of fibromyalgia according to the American College of Rheumatology [3]. (**A**) Variable importance (physician variables) in distinguishing fibromyalgia from controls, including the widespread pain index (WPI) and muscle symptoms. (**B**) Variable importance (physician variables) in distinguishing fibromyalgia from controls, excluding the Regional Pain Scale (WPI) and muscle symptoms.

**Figure 4:**
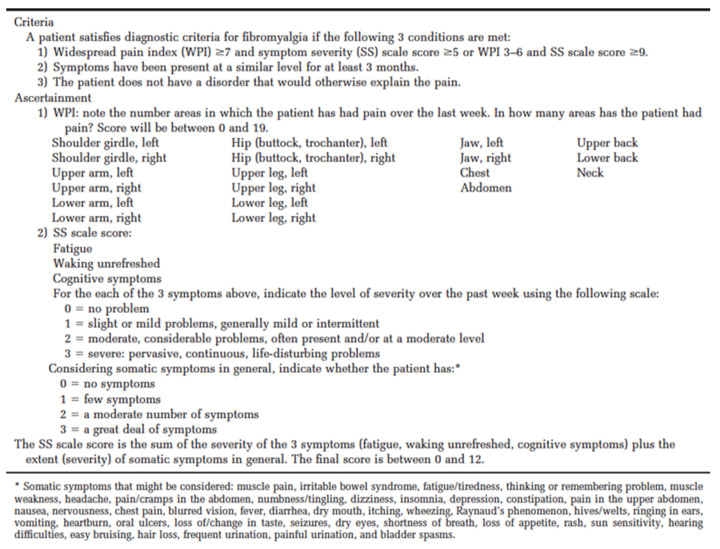
Fibromyalgia diagnostic criteria [3].

According to the American College of Rheumatology preliminary diagnostic criteria for fibromyalgia [3], muscle pain is a frequent symptom due to its recognition in multiple body areas including the chest (Figure 3 and Figure 4). The recognition of this condition is therefore foreseeable in an examination such as mammography in which the skin and pectoral muscles are compressed and stretched during its execution. The further advantage of this investigation is that it is one of the cancer screening tests for breast cancer on the entire female population and could therefore be useful in identifying a subtle pathology such as fibromyalgia at no additional cost.

We applied the same level of compression (13 daN) for all the exams we performed, so as to eliminate a possible bias in the onset of pain arousing from a different level compression. After having collected the said information, we proceeded to perform a statistical analysis to investigate the association between the above-mentioned factors and the level of pain. Our patients were informed in writing about the study’s objectives and methods and all agreed to be interviewed; the data were collected prospectively as part of a data collection for subsequent ultrasound evaluation directed at patients whose mammography were negative for malignant tumors (approval by ethic committee number 187.20).Those patients who experienced the highest level of pain (level 4) were invited to receive a rheumatological examination in order to assess a possible diagnosis of fibromyalgia along with the ACR criteria. The reason why we selected only these patients for rheumatologic examination is that they were the ones who experienced a level of pain far higher than what is reasonably expected from a standard mammography. When, one year later, these patients came back for a new roundof routine breast cancer screening (round 2), we interviewed them in order to assess how many, within this selected cluster, had received an actual diagnosis of fibromyalgia after the rheumatologic exam which we suggested.

### Statistical Analyses

Data are presented as mean and standard deviation for continuous variables and as frequency and percentages for categorical variables. Pain scores are reported as frequency (percentages) with 95% C.I. *p*-values to measure the association between each of the considered variables and pain scores were computed with a simple ordinal logistic regression model. Variables that resulted to significant to the univariate regression analysis were included in a multivariate ordinal logistic regression model. A *p*-value < 0.05 was considered statistically significant. All analyses were performed using the statistical software R, version 4.0.2.

## 3. Results

The demographic characteristics of the sample are reported in Table 1.

**Figure 5:**
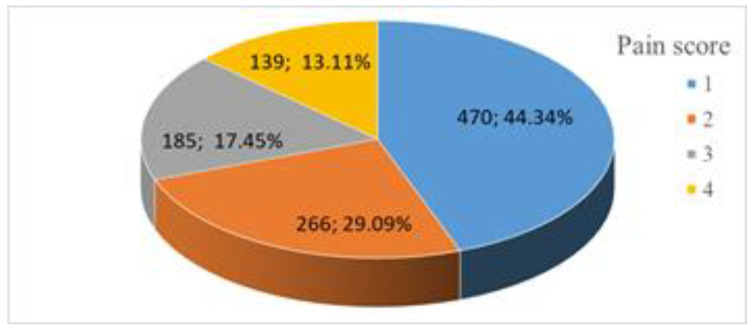
Pain score distribution.

Out of the 1060 patients who participated inour study, 470 presented level 1 pain intensity (44.34%, 41.35–47.33 CI 95%), 266 presented level 2 pain intensity (25.09%, 22.48–27.70, CI 95%), 185 presented with level 3 pain intensity (17.45% 15.17–19.74 CI 95%), 139 presented level 4 pain intensity (13.11% 11.08–15.15 CI 95%) (Figure 5); one of these patients endured such intense pain that we were unable to complete the mammography procedure; we decided not to exclude her, because for the purposes of this study, the actual completion of the screening procedure was not relevant.

Within the income groups we have divided our patients into percentiles: group 1 goes from the 1st to the 33rd percentile; group 2 goes from the 34th to the 66th percentile; and group 3 goes from the 67th to the 100th percentile. Stress level was assessed by asking our patients about their perceived stress level, similar to our pain level assessment: we felt that a more thorough assessment exceeded the goals of our study. Regarding the pain levels, we divided them into four groups based on the perceived stress level. Breast density, on the other hand, was evaluated in four classes with increasing density according to the guidelines of the ACR.

Out of the 139 women who stated a pain score of 4, 138 (one dropped out) have consulted a rheumatologist and 50 (36%, 28–44, 95% CI) were diagnosed with fibromyalgia. At ordinal logistic regression a statistically significant association (*p* < 0.01) was found between the levels pain scores and the following factors: age, coffee cups per day, number of children, stress level, breastfeeding, higher income level, breast density, benign pathology and Previous breast surgeries; there was no significant statistical association between other factors we considered and pain level (Table 2).

In particular, a medium-high level of pain (3 or 4) appears to be associated with a lower average age, number of cigarettes greater than 15, three or more children, a medium-high stress level, three or more coffees per day, having breastfed, low income, medium-high breast density and previous benign disease.

Compared to the multivariate ordinal regression model, the variables that lose significance in the multivariate model are breastfeeding, benign pathology, and previous surgery, while the level of education becomes a significant factor read in relation to the other factors. The multivariate model shows a significant goodness of fit to the data (*p*-value Pearson Chi-square coefficient < 0.01).

## 4. Discussion

The variety of clinical aspects with which pain can occur is probably related both to the possible involvement of different anatomical structures, and to the possible presence of various underlying pathophysiological conditions. In relation to the anatomical structures involved, we can distinguish pain due to skin involvement, pain due to involvement of the musculoskeletal structures and pain due to visceral involvement. Pain due to involvement of musculoskeletal structures can be due to various pathophysiological conditions such as tendonitis, polyarthritis, rheumatoid arthritis, fibromyalgia, bursitis and other painful musculoskeletal syndromes.

In the literature, the reference scales validated to diagnose pain are many [28]; we remember in particular the numeric pain rating scale (NRS) and the visual analogue scale (VAS) as one-dimensional scales, certainly simple and quick to administer but poor in information. In addition to the extent of the pain, more complex scales (e.g., McGill Pain Questionnaire (MPQ)) help to better frame not only the presence/absence of pain but also the impact on the amount of life and daily activities; there are also dedicated scales to detect the possible presence of neuropathic pain (e.g., douleur neuropatique 4 (DN4)), or the risk of addiction in patients undergoing opioid therapy (opioid risk tool (ORT), which is recently also available in Italian).

Based on what has been described, in our study we considered the expressiveness of pain in four categories.

What is most interesting, though, is that if we only consider the sample of patients who had experienced the highest level of pain while undergoing mammographic screening, the prevalence value rises to a 36%; this seems to show that assessing the level of pain during routine breast cancer procedures might be a valuable asset for identifying those patients who have a high chance of being affected by fibromyalgia.

As stated before, while fibromyalgia appears to be widespread amongst the population, diagnosis of this condition still remains elusive. Recent studies show how there is a significant degree of disagreement between international classification of diseases (ICD)-based clinical diagnosis and criteria-based diagnosis for fibromyalgia, which further contributes to complicate the diagnostic algorithm for this condition [36].

Due to such difficulties, elaborating preliminary procedures in the context of widespread and consolidated diagnostic screening paradigms can help to select those clusters of patients with an increased likelihood of being affected by such conditions and which can afterwards be redirected towards dedicated professionals for diagnosis and care.

A significant association between age, stress level, and especially breast density on one side, and pain level on the other was expected, as it is consistent with most scientific literature on the subject [37,38,39,40,41]; the correlation between these variables and the search for the disease in all pain classes will be the subject of a future study, in which the disease was only searched for within class 4.

In some studies, drinking coffee has been shown to mitigate pain perception, owing to a direct action consisting ofcentral blocking of adenosine receptors that influence pain signaling and by interaction with peripherical adenosine receptors distributed on sensory afferent fibers [42].Our results are in contrast with such findings;we may hypothesize that in our patients drinking significant amounts of coffeemight have heightened the pain perception by increasing the feeling of psychological stress, especially if they had drunk coffee right before undergoing mammographic examination, whereas patients physiologically feel anticipatory anxiety concerning the results of their screening. Further investigation will be needed to address the matter.

While a larger number of pregnancies appeared to be related to a higher pain level, previous breastfeeding did not show such a significant correlation; further studies will be needed to investigate the subject. We may postulate that having a larger number of children could be correlated with a higher stress level which is related to a higher pain level, or that hormonal changes happening during pregnancy may, somehow, be related to a lower pain feeling threshold.

Moreover, patients with a higher income and those who were more educatedexperienced a higher level of pain, although our statistical analyses offered results that were not particularly consistent regarding this subject;education to auniversity degree level, for instance, appeared to be correlated to a higher pain level in univariate analyses, but such result was not confirmed in multivariate analyses.

The most notable limit of our study is that our sample was an all-female one, which limits the applicability of our findings for both mixed gender and general population samples; also, our patients were all Caucasian.

Another limitation could be the great variability of the subjects subjected to screening: it has been shown that many factors such as the social situation, the situation and the work environment and the history of a previous injury can nevertheless influence the perception of pain and show great differences between individuals [28].

Finally, as previously said, the patients we selected for rheumatologic examination were only those who experienced the highest level of pain, as we were reasonably sure that such an intense pain was not sensibly compatible with a standard mammography. This posits an inherent limit to our study which should be taken onto account when interpreting its results.

Moreover, our study is a monocentric one and further multicentric studies may provide deeper insights on the topic.

## 5. Conclusions

Our study showed a significant association between a high level of pain while undergoing routine mammographic exam and the diagnosis of fibromyalgia; the insidious nature of such disease, on one side, and the large and widespread use of mammographic screening, on the other, suggest that the latter might be used to at least obtain a FM’s diagnosticsuspicion, until more extensive screening routines for fibromyalgia become available such as the use of advanced imaging or artificial intelligence methods already applied or being studied in the breast sector [43,44,45,46]. With few exceptions, our study confirms most scientific findings concerning factors which relate to a heightened pain perception.

## Figures and Tables

**Table 1:** Demographic characteristics of the study population.

Variable	N = 1060
Median Age (1st–3rd quartile)	54 (54–64)
Smoker	
No	847 (79.91%)
Yes	213 (20.09%)
Coffee cups (per day)	1.75 (1.35)
Children	1.79 (1.17)
Breastfeeding	
No	391 (36.89%)
Yes	669 (63.11%)
Stress	
1	253 (23.87%)
2	381 (35.94%)
3	242 (22.83%)
4	184 (17.36%)
Education	
Middle school	272 (25.66%)
High school	351 (33.11%)
Degree	411 (38.77%)
Postgraduate degree	26 (2.45%)
Income	
Group 1	797 (75.19%)
Group 2	170 (16.04%)
Group 3	93 (8.77%)
Breast Density	
ACR1	377 (35.57%)
ACR2	211 (19.91%)
ACR3	280 (26.42%)
ACR4	192 (18.11%)
Benign pathology	
No	948 (89.43%)
Yes	112 (10.56%)
Previous breast surgeries	
No	946 (89.25%)
Yes	114 (10.75%)

**Table 2:** Measured association between the pain score (from 1 to 4) and the variables age, coffee cups consumed per day, number of children, stress level, smoking habit, previous breast surgery, previous breastfeeding, the presence of benign disease, income level, education, breast density, previous benign pathology, and previous breast surgery.

		Pain Score											Multiple Model
		1		2		3		4		Total		*p*-Value	*p*-Value
**Patients**		n.	%	n.	%	n.	%	n.	%	n.	%		
		470	44.34	266	25.09	185	17.45	139	13.11	1060	100	0.000	
**Age**													
Median (25th–75th quantile)		58 (47–69)		54 (46–61)		52 (43–59)		48 (43–53)					0.000
**Number of cigarettes**												0.000	
	0–4	373	43.93	228	26.86	141	16.61	107	12.60	849	100		0.031
	05–14	59	57.28	15	14.56	19	18.45	10	9.71	103	100		0.001
	15–29	28	36.36	20	25.97	18	23.38	11	14.29	77	100		0.095
	30 or more	10	32.26	3	9.68	7	22.58	11	35.48	31	100		
**Children**												0.000	
	0	69	51.49	30	22.39	21	15.67	14	10.45	134	100		0.197
	1	148	44.31	104	31.14	40	11.98	42	12.57	334	100		0.000
	2	168	52.17	74	22.98	43	13.35	37	11.49	322	100		0.000
	3 or more	85	31.48	58	21.48	81	30.00	46	17.04	270	100		
**Stress**												0.000	
	0	151	59.68	63	24.90	39	15.42	0	0.00	253	100		0.000
	1	169	44.36	117	30.71	73	19.16	22	5.77	381	100		0.000
	2	102	42.15	54	22.31	41	16.94	45	18.60	242	100		0.000
	3	48	26.09	32	17.39	32	17.39	72	39.13	184	100		
**Coffee cups (per day)**												0.000	
	0–2	378	52.57	165	22.95	112	15.58	64	8.90	719	100		0.000
	3 or more	92	26.98	101	29.62	73	21.41	32	9.38	341	100		
**Breastfeeding**												0.000	
	No	192	49.10	127	32.48	39	9.97	33	8.44	391	100		0.132
	Yes	278	41.55	139	20.78	146	21.82	106	15.84	669	100		
**Income**												0.000	
	Group 1	348	43.66	225	28.23	141	17.69	83	10.41	797	100		0.001
	Group 2	58	34.12	33	19.41	29	17.06	50	29.41	170	100		0.000
	Group 3	64	68.82	8	8.60	15	16.13	6	6.45	93	100		
**Education**												0.451	
	Middle school	132	48.53	65	23.90	42	15.44	33	12.13	272	100		0.004
	High school	143	40.74	86	24.50	70	19.94	52	14.81	351	100		0.000
Degree or Postgraduate degree		195	44.62	115	26.32	73	16.70	54	12.36	437	100		
**Breast Density**												0.000	
	ACR1	228	60.48	96	25.46	43	11.41	10	2.65	377	100		0.000
	ACR2	104	49.29	67	31.75	23	10.90	17	8.06	211	100		0.000
	ACR3	70	25.00	74	26.43	84	30.00	52	18.57	280	100		0.760
	ACR4	68	35.42	29	15.10	35	18.23	60	31.25	192	100		
**Benign pathoogy**												0.000	
	No	441	46.52	253	26.69	147	15.51	107	11.29	948	100		0.056
	Yes	29	25.89	13	11.61	38	33.93	32	28.57	112	100		
**Previous breast surgeries**												0.002	
	No	423	44.71	249	26.32	153	16.17	121	12.79	946	100		0.513
	Yes	47	41.23	17	14.91	32	28.07	18	15.79	114	100		

## Data Availability

The data presented in this study are available on request from the 415 corresponding author. The data are not publicly available because are propriety of Università della Campania “Luigi Vanvitelli”, Napoli, Italy.
